# Enhancement of doxorubicin production in *Streptomyces peucetius* by genetic engineering and process optimization

**DOI:** 10.1186/s13568-024-01699-z

**Published:** 2024-04-24

**Authors:** Songbai Yang, Jiali Gui, Zhengyu Zhang, Jiawei Tang, Shaoxin Chen

**Affiliations:** 1 National Key Laboratory of Lead Druggability Research, Shanghai Institute of Pharmaceutical Industry, State Institute of Pharmaceutical Industry, 285 Gebaini Road, Pudong, Shanghai, 201203 P. R. China; 2 Department of Biological Medicines & Shanghai Engineering Research Center of Immunotherapeutics, Fudan University School of Pharmacy, 826 Zhangheng Road, Pudong, Shanghai, 201203 P. R. China

**Keywords:** *S. Peucetius*, Doxorubicin, Genetic engineering, Fermentation process optimization

## Abstract

Doxorubicin is an important class of anthracycline antitumor antibiotics produced by *Streptomyces peucetius*. The doxorubicin fermentation yield of the wild-type strain was very low, so it could not be produced directly by fermentation at an industrial scale due to the high cost. In the present study, *S. peucetius* SIPI-7-14 was obtained from SIPI-14 through several rounds of doxorubicin resistance screening. Then, the ketoreductase gene *dnrU* was knocked out to reduce (13*S*)-13-dihydrodaunorubicin production, and the resistance gene *drrC* was overexpressed to further enhance resistance to doxorubicin. The resulting engineered strain *S. peucetius* △U1/*drrC* produced 1128 mg/L doxorubicin, a 102.1% increase compared to that of SIPI-14. Then, fermentation medium was optimized using the response surface method. In the optimized fermentation medium, the yield of doxorubicin was increased to 1406 mg/L in shake flask on the 7th day. Furthermore, batch culture was carried out in a 10 L fermenter, and the concentration of doxorubicin reached 1461 mg/L after 7 days of culture, which was the highest yield reported to date, indicating the potential for industrial production of doxorubicin by fermentation.

**Supplementary Information:**

The online version contains supplementary material available at 10.1186/s13568-024-01699-z.

## Introduction

Doxorubicin is an anthracycline antibiotic, and its analogs include daunorubicin, epirubicin and idarubicin. As a chemical drug, doxorubicin has a broad array of anticancer activities and is used to treat various cancers, especially hemangioma and solid tumors (Mattioli et al. 2023). For most cancers, doxorubicin remains a first-line chemotherapy agent, either alone or in combination (Sritharan and Sivalingam 2021; Turinetto et al. 2023). At present, daunorubicin is produced by fermentation, followed by separation, purification and chemical synthesis to produce doxorubicin. Chemical synthesis requires side chain group protection and deprotection, polluting the environment and having high production costs. In 1969, it was first reported that doxorubicin is produced in *S. peucetius* ATCC27952 by fermentation (Arcamone et al. 1969). The use of doxorubicin directly obtained by fermentation is limited by the low titer used during fermentation. If a large amount of doxorubicin can be obtained directly by fermentation through a high-yielding strain and by optimizing the fermentation process, this approach will be an environmentally friendly and efficient method.

The biosynthetic pathway of doxorubicin can be divided into three main pathways (Gao et al. 2022; Hutchinson and Colombo 1999). The synthesis of ɛ-rhodomycinone is the first pathway involved. Under the action of a type II polyketide synthase (PKS) encoded by the *dpsABCDGEFY* genes, 12-deoxyacranic acid is synthesized by a multistep reaction with propionyl CoA as the starting unit and malonyl CoA as the extension unit. Then, the ɛ-rhodomycinone aglycone is produced by oxygenase, methyltransferase, cyclization and reductase. The second synthesis pathway of doxorubicin is that of TDP-L-daunosamine. Through a six-step reaction, D-glucose-1-phosphate from the central metabolic pathway is synthesized to produce TDP-L-daunosamine, which involves a cluster of *dnm* genes, including *dnmL*, *dnmM*, *dnmU*, *dnmT*, *dnmJ*, and *dnmV*. Finally, glycosylation and postmodification of daunorubicin and doxorubicin occur. Rhodomycin-D is produced by ɛ-rhodomycinone and TDP-L-daurosamine through the action of glycosyltransferase. After postmodification (demethylation, decarboxylation and hydroxylation), daunorubicin and doxorubicin are obtained. In the branch pathway, daunorubicin is converted to 13(S)-13-dihydrodaunorubicin via catalysis by the enzyme encoded by the *dnrU gene*. Moreover, daunorubicin and doxorubicin are converted into acid-sensitive baumycin by the enzymes encoded by the genes *dnrH* and *dnrX*, as shown in Additional file: Fig. S1.

In *S. peucetitus* ATCC 27,952, the yield of doxorubicin is low, making its large-scale production difficult and high-cost. To address this problem, several efforts have been made to increase the production of doxorubicin by genetic engineering. Based on the biosynthetic pathway of doxorubicin, its production may be limited by several key enzymes, such as glycosylated genes and a P450 enzyme DoxA. Therefore, they were engineered to increase the fermentation yield of doxorubicin. The overexpression of glycosylated genes has been utilized to increase the fermentation yield of doxorubicin. Malla (Malla et al. 2009) achieved a 5.6-fold increase in doxorubicin production (approximately 10 mg/L) by simultaneously overexpressing the gene glycosyltransferase *dnrS/dnrQ* and the sugar synthesis pathway gene *desIII/desIV*. Recently, Zhang (Zhang et al. 2023a) obtained a DoxA mutant, DoxA(P88Y), showed a 56% increase in the conversion efficiency of daunorubicin to doxorubicin. These results indicated that some enzymes are limiting factors in the biosynthetic pathway and their engineering is an important approach to improve doxorubicin production. In addition, it was reported that the synthesis of the secondary metabolite doxorubicin is tightly regulated. The fermentation yield of doxorubicin was increased by disrupting negative regulatory genes and overexpressing positive regulatory genes. Noh(Noh et al. 2010)enhanced the doxorubicin fermentation yield by approximately 35% (approximately 75 mg/L) by disrupting the downregulation of the gene *wblA*. In addition, the expression of the *AfsR* homologous regulatory gene (*AfsR-p*) in *S. peucetius* ATCC27952 led to a 4-fold increase in doxorubicin production (approximately 80 mg/L) (Parajuli et al. 2005). ParK (Park et al. 2005) increased the fermentation yield of doxorubicin by 30.8-fold (approximately 11 mg/L) through the overexpression of the regulatory gene *dnrI* in a high-copy-number plasmid combined with the optimization of the medium. Moreover, Malla(Malla et al. 2010a) increased the fermentation yield of doxorubicin by 4.3 times (approximately 7 mg/L) by simultaneously overexpressing the regulatory gene *dnrN*-*dnrI*-*afsR*. However, in all these studies, improving the yield of doxorubicin were focused on wild-type strains with low doxorubicin yields. Therefore, genetic modification of high-yielding strains is important for industrial application.

In our previous study, we generated a *S. peucetius* SIPI-14 strain with significantly improved doxorubicin levels by combining the traditional method of UV and ARTP mutagenesis (Wang et al. 2018). In the present study, a strain with doxorubicin resistance was obtained from SIPI-14 for further genetic modification combined with fermentation medium optimization to significantly improve the yield of doxorubicin, which laid a foundation for the large-scale industrial production of doxorubicin by direct fermentation in the future.

## Materials and methods

### Strains, plasmids and primers

The strains and plasmids used in this research are listed in Table 1, and the primers used are listed in Additional file: Table S1.

**Table 1:** Strains and plasmids used in this study

Strains or plasmids	Relevant characteristic	Source
**Strains**		
*S. peucetius* ATCC 27,952	Doxorubicin-producing strain	ATCC
*S. peucetius* SIPI-14	Doxorubicin-producing mutant strain derived from the *S. peucetius* ATCC 27,952	(Wang et al. 2018)
*S. peucetius* SIPI-7-14	Doxorubicin-producing mutant strain derived from the *S. peucetius* SIPI-14	This study
△U1	Knockout gene *dnrU* in *S. peucetius* SIPI-7-14	This study
△H4	Knockout gene *dnrH* in *S. peucetius* SIPI-7-14	This study
△X1	Knockout gene *dnrX* in *S. peucetius* SIPI-7-14	This study
△U1/*drrC*	Overexpressed gene *drrC* in △U1	This study
△U1/*drrAB*	Overexpressed gene *drrAB* in △U1	This study
△U1/*drrD*	Overexpressed gene *drrD* in △U1	This study
△U1/*drrABC*	Overexpressed gene *drrAB* and gene *drrC* in △U1	This study
DH5α	*F–Φ80lacZΔM15 Δ(lacZYA-argF) U169 deoR recA1 endA1 hsdR17(rk-mk+) phoA* *supE44 λ-thi-1 gyrA96 relA1*	CWBIO, China
S17-1	*RP4-2 (Km::Tn7,Tc::Mu-1), pro-82, LAMpir, recA1, endA1, thiE1, hsdR17, creC510*	WEIDI, China
**Plasmids**	Relevant characteristic	Source
pSET152	*E. coli*–Streptomyces shuttle vector containing oriT, ΦC31 integration site (attP), aac (3) IV and PermE*, Apr^r^	(Bierman et al. 1992)
pSET	pSET152 plasmid with deletion of φC31 integration site, used for gene knockout, Apr^r^	(Li et al. 2022)
pSET-△*dnrU*	pSET with two homologous arms flanking from *dnrU*	This study
pSET-△*dnrH*	pSET with two homologous arms flanking from *dnrH*	This study
pSET-△*dnrX*	pSET with two homologous arms flanking from *dnrX*	This study
pSET152-*drrC*	pSET152 with *drrC* inserted *Nde*I, under the control of the constitutive promoter PermE*	This study
pSET152-drr*AB*	pSET152 with *drrAB* inserted *Nde*I, under the control of the constitutive promoter PermE*	This study
pSET152-drr*ABC*	pSET152-*drrC* with *drrAB* inserted *Spe*I under the control of the constitutive promoter PermE*	This study
pSET152-*drrD*	pSET152 with *drrD* inserted *Nde*I, under the control of the constitutive promoter PermE*	This study

### Culture of *S. peucetius* in shake flasks

*S. peucetius* was cultured in SP slant medium (Maltodextrin, 2.0%; yeast extract, 1.5%; enzymatic hydrolysis of casein, 0.5%; glucose, 0.4%; Soy peptone, 0.25%; KH_2_PO_4_, 0.15%; CaCO_3_, 0.2%; agar, 2.0%; pH 7.2) at 28 °C for 4–6 days, after which the strain (approximately 1.0 cm × 1.5 cm mycelia) was picked and inoculated into a 250 mL shake flask containing 25 mL of seed medium (corn starch, 0.5%; glucose, 0.5%; soybean flour, 3.0%; dry yeast powder, 0.1%; NaCl, 0.1%; KH_2_PO_4_, 0.1%; MgSO_4_.7H_2_O, 0.1%; pH 7.2) and subsequently grown at 28 °C for 40–46 h. Afterward, 2 mL of the seed culture was transferred to a 250 mL shake flask containing 25 mL of fermentation medium (soybean oil, 8.0%; soybean meal, 3.0%; NaCl, 0.2%; CaCO_3_, 0.3%; pH 6.2). Fermentation shake flasks were incubated at 28 °C in a rotatory shaker for 6 days.

### Culture of *S. Peucetius* in a 10 L fermenter

The 1.0 cm × 1.5 cm mycelia of the engineered *S. peucetius* △U1/*drrC* were picked and cultured in a seed medium at 28 ℃ for 42–48 h at 200 rpm. 5% seed cultures were transferred to a 10 L bioreactor containing 6 L of fermentation medium. The incubation temperature, initial pH and aeration conditions were 28 ℃, 6.2 and 0.03–0.06 vvm (air volume/culture volume/min), respectively. The dissolved oxygen was automatically associated with agitation, which was controlled at 30-35% during fermentation. The yield of doxorubicin was measured at regular intervals for 8 days.

### Screening of *S. peucetius* with doxorubicin resistance

The *S. peucetius* SIPI-7-14 cultured on the plate were inoculated into a seed medium containing 50 mg/L doxorubicin at 28 ℃ and 220 rpm for 2 days. The seed culture mixture was continuously transferred to fresh seed medium 2 times, diluted on a solid plate to obtain single colonies, and cultured for doxorubicin production in shake flasks. The strain with the highest yield of doxorubicin was selected for the next round of screening. The concentration of doxorubicin in the seed medium was gradually increased from 50 mg/L to 100 mg/L and 150 mg/L to obtain doxorubicin-resistant strains.

### Intergeneric conjugal transfer of plasmid from *E. Coli* to *S. Peucetius*

*S. peucetius* stored in 20% glycerol at -80 °C was spread onto SP plates and cultured in an incubator at 28 °C for 4–6 days. Mycelia (1.0 cm × 1.5 cm) were picked and inoculated into seed media for 2 days, after which 10% of the inoculum was transferred to tryptic soy broth (TSB) media for culture for 20–24 h. *E. coli* S17-1 was cultured overnight at 37 °C in 5 mL LB tubes, after which 2% of the inoculum was cultured in 50 mL LB shake flasks. When the OD of *E. coli* reached 0.4–0.6, mixed cultures of *Streptomyces* and *E. coli* were incubated in RP media (soluble starch, 2.0%; soybean powder, 1.0%; valine, 0.05%; NaCl, 0.2%; KH_2_PO_4_, 0.05%; MgSO_4_.7H_2_O, 0.1%; MgCl_2_.6H_2_O, 1.6%; agar, 2.0%; CaCO_3_, 0.3%). The plates were incubated at 28 °C for 18–20 h and overlaid with 1 mL of sterile water containing 150 mg/L nalidixic acid and 200 mg/L apramycin. These plates were further incubated in a 28 °C incubator for 5–7 days, after which apramycin-resistant colonies were picked for PCR validation.

### Strain construction

The knockout strains △U1, △H4 and △X1 were derived from SIPI-7-14 with the deletion of a 351 bp, 783 bp and 1221 bp fragment on the gene *dnrU*, *dnrH* and *dnrX*, respectively. The constructed △U1 knockout mutant strain was used as an example. The *S. peucetius* SIPI-7-14 genome was used as a template to obtain the 1121 bp upstream and 1450 bp downstream homology arms of *dnrU* by PCR amplification with the primers *dnrU*-UP-F/R and *dnrU*-DOWN-F/R. The upstream and downstream homology arms were fused together by overlapping PCR with the primers *dnrU*-UP-F/*dnrU*-DOWN-R. The PCR product was digested with *Hind*III/*Xba*I and then assembled on the pSET vector to construct the plasmid pSET-△*dnrU* (Additional file: Fig. S2a), which was used to delete the *dnrU* gene. The well-established plasmid was subsequently transformed into S17-1, which was subsequently conjugated with strain SIPI-7-14. The colonies that were apramycin resistant were regarded as the integrating mutants, in which the first homologous single-crossover occurred. The resulting mutants were then subcultured six times in TSB medium without the antibiotic apramycin and diluted to spread on SP plates. The colonies that were phenotypically sensitive to apramycin were selected. The primers Δ*dnrU* F/R were used for colony PCR verification. The PCR products were subsequently sequenced by GENEWIZ for verification, and all primers were synthesized by Jie Li Biology (Li et al. 2022). The mutant strains ΔH4 and ΔX1 were constructed using the same method as that used to construct ΔU1. Among them, the two homology arms of the mutant strains △H4 and △X1 were obtained from the SIPI-7-14 genome using the primer pairs *dnrH*-up-F/R and *dnrH*-down-F/R and *dnrX*-up-F/R and *dnrX*-down-F/R, respectively. The constructed plasmids pSET-△*dnrH* and pSET-△*dnrX* were shown in Additional file: Fig. S2b and Fig. S2c. The final verification PCR primers used were Δ*dnrH* F/R and Δ*dnrX* F/R. The PCR products of the △*dnrH* and △*dnrX* genes were validated by further Sanger sequencing.

To overexpress the *drrC* gene, the *drrC* gene fragment was first obtained from the *S. peucetius* SIPI-7-14 genome by PCR using the primers *drrC* F/R. The plasmid pSET152 was subsequently digested with *Nde*I and ligated to the PCR product to construct the integrated plasmid pSET152-*drrC* (Additional file: Fig. S2d). The correctly sequenced plasmid was introduced into S17-1 competent cells. The *drrC* gene was subsequently integrated into *S. peucetius* △U1 by conjugative transfer. The plasmids pSET152-*drrAB* (Additional file: Fig. S2e) and pSET152-*drrD* (Additional file: Fig. S2f) were constructed in the same way. The *drrAB* and *drrD* genes were obtained by PCR amplification using the primers *drrAB* F/R and *drrD* F/R, respectively. The target gene *drrAB* was amplified from the *S. peucetius* SIPI-7-14 genome using the primers *drrAB* F (TY)/R. The plasmid pSET152-*drrC* was subsequently digested with *spe*I and ligated to the PCR product to construct the integrated plasmid pSET152-*drrABC* (Additional file: Fig. S2g). The genetically engineered strains containing the overexpressed genes were cultured in shake flasks, and the yield of doxorubicin was analyzed using HPLC. Genes with accession numbers were as follow: *drrA* (Accession number: PP049447), *drrB* (Accession number: PP049448), *drrC* (Accession number: L76359) and *drrD* (Accession number: PP049449).

### Optimization of fermentation medium by RSM

The fermentation medium was optimized by response surface methodology (RSM) using the statistical design software Box‒Behnken design (BBD), version 8.06. Three factors, (A) maltodextrin, (B) dry yeast powder, and (C) calcium chloride, were used, and all the variables consisted of three levels (-1, 0, and 1), representing minimum, intermediate, and maximum values, respectively (as shown in Additional file: Table S2). The seeds were cultured for 40–46 h. The engineered strain △U1/*drrC* was selected for fermentation in shake flasks. 10% of the seeds cultured for 40–46 h were transferred to fermentation media for 6 days. Each experiment was repeated in three parallel shake flasks. The fermentation yield of doxorubicin was measured by HPLC.

### Analysis of cell growth and doxorubicin production

For biomass determination, 4 mL of fermentation broth was centrifuged at 12,000 rpm for 15 min. Biomass (%) = [(F − S)/F] × 100%, where F represents the volume of fermentation broth and S represents the volume of supernatant.

The fermentation broth was adjusted to pH 1.5–1.8 with 6 mol/L HCl, and 0.1 mL of the fermentation broth was subsequently added to 0.9 mL of ethanol, mixed and sonicated for 30 min at 4 Hz. After centrifugation at 12,000 rpm for 10 min, the supernatant was filtered through a 0.22 μm membrane, after which the samples were detected via Agilent 1260 high-performance liquid chromatography (HPLC). The chromatographic column used was an Agilent SB-18 (3.5 μm, 4.6 × 250 mm), the mobile phase was used at a flow rate of 1 mL/min, the detection wavelength was 254 nm, and the column temperature was 30 ℃. The mobile phase was composed of buffer: acetonitrile: methanol = 500:500:60, where the buffer contained 0.68 mL of phosphoric acid and 1.44 g of sodium dodecyl sulfate in 500 mL of purified water.

### Analysis of transcriptional levels by quantitative real-time PCR

For the control strain *S. peucetius* ΔU1 and the overexpression strain ΔU1/*drrC*, 20 mL of fermentation broth was collected on the 4th and 6th days, respectively, and centrifuged at 6000 rpm for 10 min at 4 °C to collect the cells. Total RNA was extracted according to the experimental manual provided with the Ultrapure RNA Kit (CWBIO, Beijing, China). Before reverse transcription of the RNA into cDNA, residual DNA in the RNA samples was removed using RNase-free DNase I (Takara, Dalian, China). Reverse transcription was performed using the Kit PrimeScript™ RT Reagent Kit (Takara, Dalian, China) according to the method provided by the kit manufacturer. The obtained cDNA was used as a template for qPCR, and the transcription level of the *drrC* gene was determined with the *1*6S *rRNA* gene as the reference gene. The primers used for qPCR amplification of the *drrC* gene *and 1*6S *rRNA* gene were Q*drrC* F/R and 16 S RNA F/R, respectively (Additional file 1: Table S1). The qPCR amplification conditions included 50 °C for 2 min and 95 °C for 10 min; 40 cycles of 95 °C for 15 s and 60 °C for 60 s; and a final step of 95 °C for 15 s. Changes in the fold change in gene expression levels were quantified using the 2^− *ρρCt*^ method (Livak and Schmittgen 2001). Each qRT‒PCR experiment was repeated three times.

### Statistical analysis

Each experiment in this study had been replicated three times, with the error bars showing the standard deviations. Statistical analyses involving comparison between the test data and the control date were performed with t-student test (*P* < 0.001) and one-way ANOVA by using GraphPad Prism Version 8.0 software and Origin Version 8.5 software.

## Results

### Doxorubicin resistance screening

**Fig. 1:**
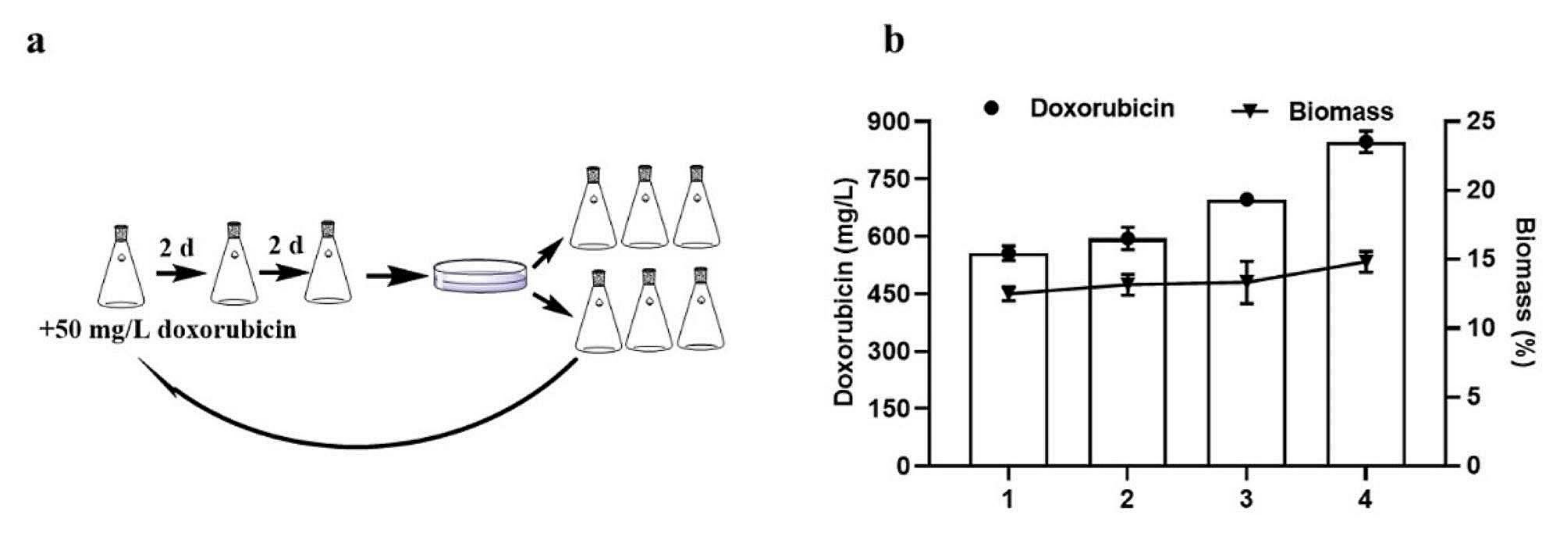
Improvement of doxorubicin yield by resistance screening **a** Fifty milligrams/L doxorubicin was added to the seed medium for 2 days of culture, after which 10% of the inoculum was transferred to fresh seed medium 2 times. The seed culture mixture was diluted and spread onto a plate, and single colonies were selected for shake flask fermentation. The strain with the highest yield was selected for the next round of screening. **b** Doxorubicin-producing *S. peucetius* were screened under different conditions and verified by shake flask culture: (1) natural selection, (2) seed solution with 50 mg/L doxorubicin, (3) seed solution with 100 mg/L doxorubicin, and (4) seed solution with 150 mg/L doxorubicin. The error bars are the standard deviations of three replicates

*S. peucetius* SIPI-14, which has significantly increased doxorubicin levels, was generated by traditional mutagenesis in our study (Wang et al. 2018). Due to its strong cytotoxicity, doxorubicin has a strong inhibitory effect on the growth of *S. peucetius* strains themselves. In this study, doxorubicin was added to the seed medium to obtain strains with greater doxorubicin resistance through continuous resistance pressure screening. Initially, 50 mg/L doxorubicin was added to the liquid medium. After continuous cultivation, the concentration of doxorubicin in the seed medium was gradually increased from 50 mg/L to 150 mg/L to screen for high-yielding strains that could grow in a resistant environment (Fig. 1a). Through continuous doxorubicin resistance screening, the biomass of the strains gradually increased from 12.5 to 14.8%, and the ability to produce doxorubicin was significantly enhanced. The high-yielding strain SIPI-7-14 was selected from seed medium containing 150 mg/L doxorubicin. The yield of doxorubicin reached 847 mg/L, which was 51.7% greater than that of SIPI-14 (Fig. 1b).

### Construction of the *dnrU*, *dnrH* and *dnrX* gene knockout strains

**Fig. 2:**
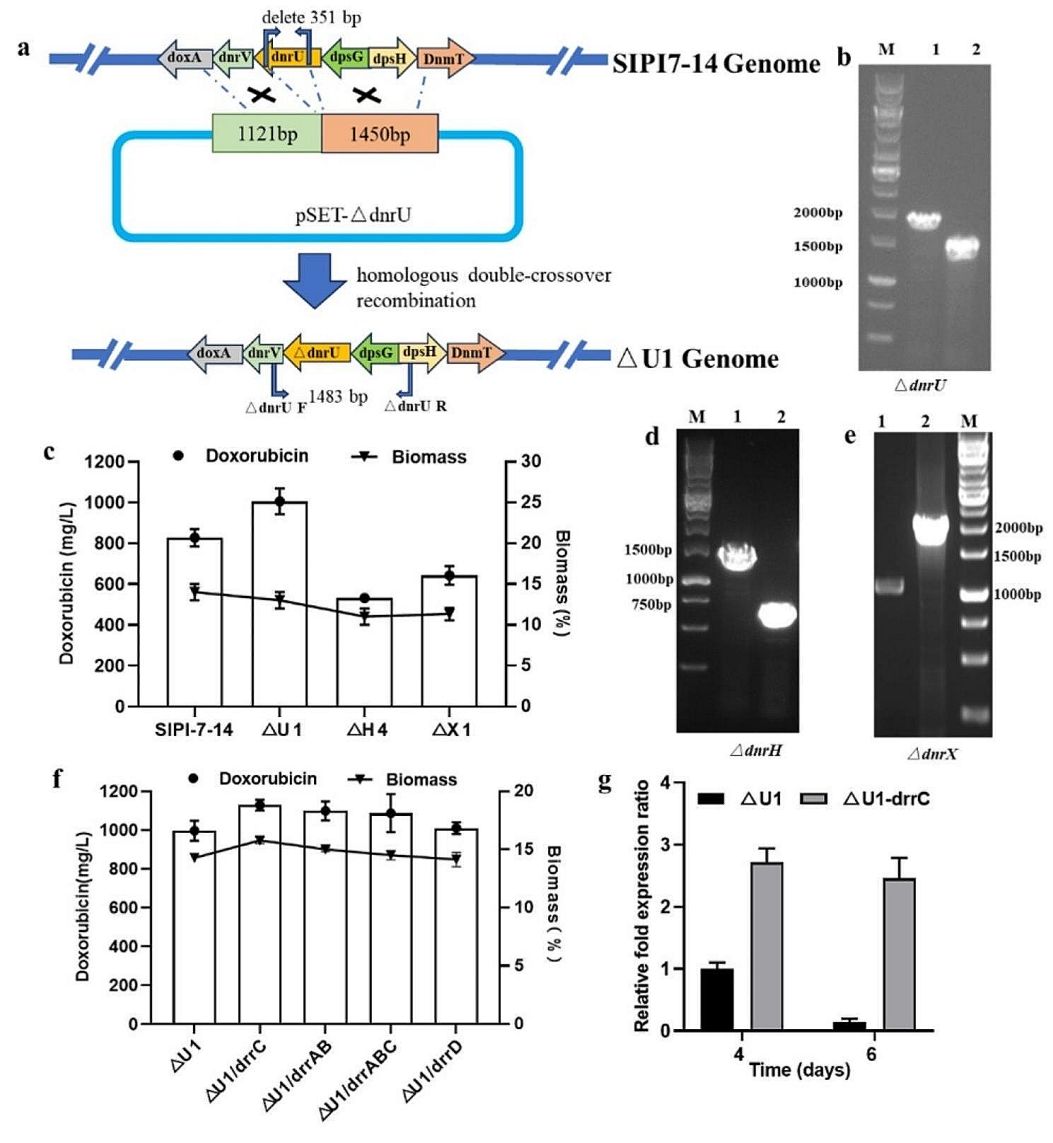
Inactivation of competing pathway genes and overexpression of the resistance genes **a** Schematic representation of the disruption of the target gene *dnrU* in *S. peucetius*. **b** The parental strain SIPI-7-14 and the engineered strain △U1 were verified by PCR. M: marker, 1: control (SIPI-7-14), 2: the engineered strain △U1 (351 bp in the knockout gene *dnrU* in the genome of strain SIPI-7-14). **c** Comparison of doxorubicin production and biomass by the SIPI-7-14 strain and the engineered strains △U1, △H4 and △X1 in shake flasks after 6 days of fermentation. The error bars indicate the standard deviations. **d** The parental strain SIPI-7-14 and the engineered strain △H4 were verified by PCR. M: marker, 1: control (SIPI-7-14), 2: the engineered strain △H4 (783 bp in the knockout gene *dnrH* in the genome of strain SIPI-7-14). **e** The parental strain SIPI-7-14 and the engineered strain △X1 were verified by PCR. M: marker, 1: control (SIPI-7-14), 2: the engineered strain △X1 (1221 bp in the knockout gene *dnrX* in the genome of strain SIPI-7-14). **f** The engineered strains △U1/*drrC*, △U1/*drrAB*, △U1/*drrABC* and △U1/*drrD* were generated by overexpressing the resistance genes *drrC*, *drrAB*, *drrABC* and *drrD* in the parental strain △U1, respectively, and were subsequently verified by fermentation in shake flasks to determine their doxorubicin production and biomass. Three parallel shakers were performed for each strain. **g** The transcription levels of the *drrC* gene were compared between △U1/*drrC* and △U1 by using shake flask fermentation broth collected on the 4th and 6th days. The error bars indicate the standard deviations

Through sequential doxorubicin resistance screening, it is difficult to further improve the yield of doxorubicin. Therefore, genetic engineering was selected for strain modification. After producing daunorubicin and doxorubicin, the strains could also produce byproducts. According to the branch biosynthetic pathway of doxorubicin shown in Additional file: Fig. S1, the *dnrU* gene in the branch metabolic pathway encodes a ketoreductase that catalyzes the reduction of the intermediate daunorubicin at position C-13 to form 13-dihydrodaunorubicin. The genes *dnrH* and *dnrX* encode enzymes that catalyze the synthesis of baumycin-like glycosides from daunorubicin and doxorubicin. In this study, homologous double-crossover recombination was used to knock out the 351 bp fragment of *dnrU* (Fig. 2a) to obtain the ΔU1 mutant. The parent strain SIPI-7-14 and the ΔU1 mutant were confirmed by PCR (Fig. 2b). △H4 and △X1 were obtained using the same method by deleting *dnrH* and *dnrX* (Fig. 2d and e), respectively. The biomass of the engineered strain △U1 decreased slightly compared to that of the parental strain SIPI-7-14, from 15 to 14%. However, the yield of doxorubicin produced by the engineered strain △U1 was 1005 mg/L, which was 21.5% greater than that produced by the parental strain. However, when the branch pathway genes *dnrH* and *dnrX* were knocked out, the yield of doxorubicin in the knockout strains △H4 and △X1 decreased (Fig. 2c).

### Overexpression of the resistance genes *drrC*, *drrAB*, *drrD* or *drrABC* increases doxorubicin production

Antibiotic-producing actinomycetes maintain self-resistance through the presence of resistance genes. In contrast to the ATP-dependent efflux pump mechanism composed of *drrA* and *drrB* (Kaur 1997; Li et al. 2014), the *drrC* gene encodes a UvrA-like protein that binds to DNA and can remove daunorubicin from the insertion site, thus enabling actinomycetes to tolerate their own products (Lomovskaya et al. 1996; Prija and Prasad 2017). The integrated plasmid pSET152 was used to overexpress the *drrC*, *drrAB* and *drrD* genes in the strain △U1 to obtain the engineered strains △U1/*drrC*, △U1/*drrAB* and △U1/*drrD*, respectively. The engineered strains were verified by shake flask fermentation. The doxorubicin yield of the engineered strain △U1/*drrC* was 13.2% greater than that of the parental strain △U1, reaching 1128 mg/L (Fig. 2f). The engineered strain △U1/*drrAB* produced 1098 mg/L doxorubicin. However, the engineered strain △U1/*drrD* produced 1010 mg/L doxorubicin in a shake flask, which was almost the same as that of the parental strain. Furthermore, the *drrAB* and *drrC* genes were overexpressed in the △U1 strain to obtain the engineered strain △U1/*drrABC*. However, the combined overexpression of the *drrAB* and *drrC* genes resulted in a lower fermentation yield than the single expression of *drrC*. In conclusion, the engineered strain △U1/*drrC* produced the highest fermentation yield in shake flasks. The transcription levels of the *drrC* gene in the engineered strain △U1/*drrC* and the parental strain △U1 were subsequently analyzed. The transcription level of *drrC* in △U1 decreased significantly during the culture process. The transcription level of *drrC* in the strain △U1/drrC was significantly greater than that in the △U1 strain (Fig. 2g). A high level of *drrC* expression increases the tolerance of the strain to high concentrations of doxorubicin, resulting in maintenance of the biomass and the production of additional products.

### Fermentation medium optimization by RSM

**Fig. 3:**
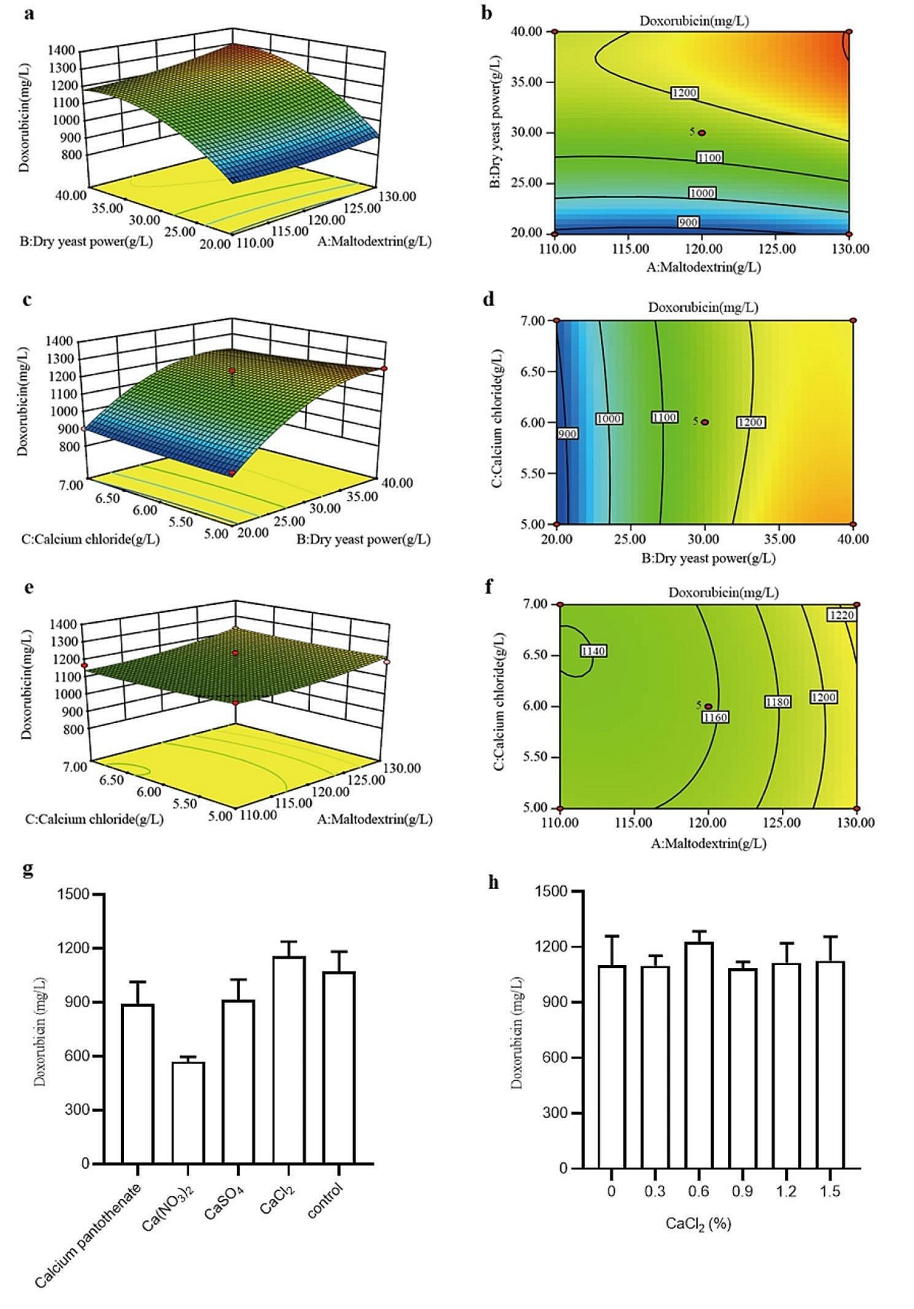
Optimization of fermentation medium optimization by RSM. **a, b** Effects of dry yeast power and maltodextrin on the doxorubicin yield. **c, d** Effects of dry yeast power and calcium chloride on the doxorubicin yield. **e, f** Effects of calcium chloride and maltodextrin on the doxorubicin yield. **g** Effect of different calcium sources on doxorubicin production. **h** Effect of the calcium chloride concentration on doxorubicin production

In our previous studies, Wang (Wang et al. 2018) found that maltodextrin and dry yeast powder had a positive effect on doxorubicin by using single-factor experiments. In addition to maltodextrin and dry yeast powder, we also found that calcium salts affect the synthesis of doxorubicin. The effects of four different calcium sources, calcium pantothenate, Ca(NO_3_)_2_, CaSO_4_ and CaCl_2_, on the yield of doxorubicin fermentation were studied, and CaCl_2_ was shown to improve the production of doxorubicin (Fig. 3g). Therefore, *S. peucetius* △U1/*drrC* cultured in fermentation media supplemented with different concentrations of CaCl_2_ were investigated in shake flasks. When the concentration of CaCl_2_ was 0.6%, the yield of doxorubicin increased by 11.3%, reaching 1228 mg/L (Fig. 3h). The components containing maltodextrin, yeast powder and calcium chloride were further optimized by the response surface method.

For the response surface optimization experiment, a total of 17 formulations were designed (as shown in Additional file: Table S3), each with three parallel shake flasks. The center points were the same for 5 points and different for the remaining 12 points. Doxorubicin yields ranging from 921 to 1333 mg/L were observed in 17 experiments with three factors and three levels (Additional file: Table S3). The response of doxorubicin content was expressed by a second-order polynomial equation, which was obtained by multiple regression analysis (R^2^ = 0.9548 and *p* < 0.0006):

Here, Y represents the doxorubicin yield. A, B and C represent maltodextrin, dry yeast powder and CaCl_2_, respectively. ANOVA was used in the response surface model to verify the reliability of the statistical model and the statistical significance of the parameters, as described in Additional file: Table S4. The p value of the second-order PRS model is 0.0006, indicating that this model is significant. In addition, lack of fit was used to evaluate model reliability, and a nonsignificant lack of fit was satisfactory. In this model, the lack of fit F value is 0.69, which is insignificant relative to the pure error, indicating that the model is suitable. The predicted R^2^ is predicted by the response surface model. The difference between the adjusted R^2^ and the predicted R^2^ was within 0.2, indicating that the model was valid. In this way, Pred R^2^ = 0.7063 and Adj R^2^ = 0.8966 are in line with the requirements, indicating that the experimental results are reliable.

The 3D response surface plots and the 2D contour plots represent the prediction equations in Fig. 3a-f. The best predictive values were 130 g/L maltodextrin, 39 g/L dry yeast powder and 5 g/L calcium chloride.

**Fig. 4:**
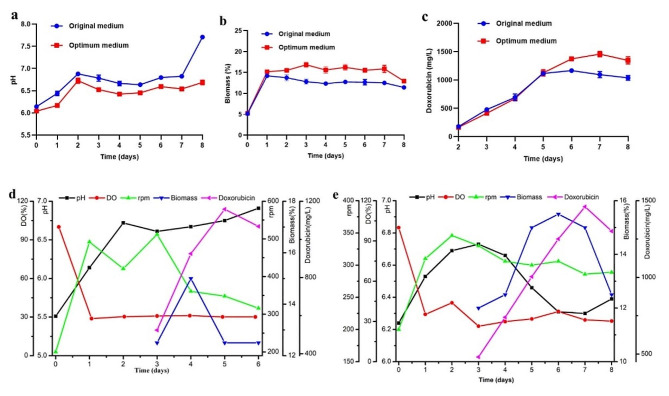
Comparison of the fermentation process of △U1/drrC in shake flasks and 10-L fermenter. **a** Changes in pH, **b** changes in biomass, and **c** production of doxorubicin in the original formulation and in the optimized formulation in shake flasks, respectively. The fermentation process of △U1/*drrC ***d** in the original formulation and **e** in the optimized formulation in a 10-L fermenter

The optimal fermentation parameters were predicted to be 130 g/L maltodextrin, 39 g/L dry yeast powder, 5 g/L CaCl_2_, 2 g/L NaCl, and 3 g/L CaCO_3_. △U1/*drrC* was cultured in the original formulation or the optimized formulation in shake flasks to compare the differences in doxorubicin yield, biomass and pH (Fig. 4a-c).

There was no significant difference in the fermentation yield of △U1/*drrC* between the optimized formula and the original formula during the five days of shaking flask fermentation. However, with the optimized formula, △U1/*drrC* produced more products after the 6th day, and the highest yield of doxorubicin was 1406 mg/L on the 7th day (Fig. 4c). On the 6th day, the yield of doxorubicin reached 1340 mg/L (Fig. 4c), which was very close to the model-predicted value of 1323 mg/L, indicating that the prediction model was effective. The optimized formulation was more suitable for the growth of △U1/*drrC*; Thus, having more biomass resulted in a greater doxorubicin yield (Fig. 4b). In addition, the pH of the optimized formulation was maintained between 6.4 and 6.6, which was lower than that of the original formulation, indicating that the strain underwent a slower aging reaction in the new medium than in the other media, thereby increasing the fermentation period (Fig. 4a).

### Batch culture of △U1/*drrC* in 10 L fermenter for doxorubicin production

To further expand the scale of cultivation, fermentation was carried out in a 10 L fermenter using the original and optimized media with *S. peucetius* △U1/*drrC* (Fig. 4d and e). In the original formulation, the agitation increased rapidly during the first three days, at which point the strain grew rapidly. The biomass of the strain reached a maximum of 15% on the 4th day. The titer of doxorubicin reached 1158 mg/L on the 5th day of fermentation. During fermentation, the trend of the change in pH first increased, then decreased, and finally increased again. The fermentation period was short, and the biomass decreased rapidly (Fig. 4d). With the optimized formula, the growth rate increased rapidly in the first two days, the strains grew rapidly, and the biomass was slightly greater than that of the original formulation, reaching 15.5%; However, the biomass was maintained for a longer time. The trend of the pH change in the optimized formulation was consistent with that in the original formulation, but the pH was maintained at approximately 6.4 during the later period, which was lower than that of the original formulation. The duration of doxorubicin production in the optimized formulation was longer, probably because the biomass in the optimized formulation was maintained for a longer time, and a lower pH was more favorable for strain production. The yield of doxorubicin reached a maximum of 1461 mg/L on the 7th day (Fig. 4e), which was slightly greater than the yield of the shaker culture.

## Discussion

Secondary metabolites are generally toxic to their producers. The cytotoxicity of doxorubicin can inhibit the growth of *S. peucetius* and hinder product production. In our study, *S. peucetius* was continuously cultivated in seed medium containing doxorubicin, which made the strains more tolerant to the toxicity of their own products. By adaptive evolution, the resulted *S. peucetius* SIPI-7-14 showed higher biomass and doxorubicin yield than that of *S. peucetius* SIPI-14. The high-yielding strains can be used as better cell factories for doxorubicin production. To further increase the product tolerance, overexpression of resistance genes is another widely applied approach. Cellular resistance in *S. peucetius* is mediated by genes, including *drrA*, *drrB*, *drrC* and *drrD* (Karuppasamy et al. 2015). An efflux pump composed of the proteins *drrA* and *drrB* removes doxorubicin from cells (Kaur 1997). Like UvrA, the protein drrC may recognize and eliminate daunorubicin, which is embedded in DNA, thus allowing DNA replication and transcription to continue (Prija and Prasad 2017). Expression of *drrC* in the *Escherichia coli uvrA* strain can significantly increase the daunorubicin resistance of the strain (Lomovskaya et al. 1996). Directly downstream of *drrA* and *drrB* lies *drrD*, which is involved in self-resistance as well. *S. peucetius drrD* deletion mutant showed reduced self-resistance, but the molecular mechanism has not yet been known (Karuppasamy et al. 2015). In our study, overexpression of *drrA* and *drrB* resulted in an increase in the production of doxorubicin. In addition, overexpression of the resistance gene *drrC* produced 1128 mg/L doxorubicin, a 102.1% increase compared to that of SIPI-14. However, the combination of the genes *drrA*, *drrB* and *drrC* did not result in a greater yield than did the overexpression of *drrC* alone. This phenomenon is consistent with the findings of previous reports in the literature, in which the expression of both resistance systems was shown to be worse than the expression of an individual resistance system (Malla et al. 2010b). We have also tried to overexpress *drrD* in △U1, but the strain showed almost the same productivity as the parental strain.

Secondary metabolites can generally be enhanced by deleting genes of competing pathways. In the pathway of doxorubicin biosynthesis, the *dnrU* coding product can catalyze daunorubicin to produce byproducts, while the *dnrH* and *dnrX* coding products can use daunorubicin and doxorubicin to produce baumycin analogs. It was reported that in *S. peucetius* 29,050, blocking the *dnrU* and *dnrX* increased the yield of doxorubicin by three times, compared with that of the parent strain. Inactivation of *dnrH* led to an 8.5-fold increase in daunorubicin production and doxorubicin production also increased (Lomovskaya et al. 1998, 1999; Scotti and Hutchinson 1996). In this study, gene *dnrU* was disrupted in the *S. peucetius*s SIPI-7-14 and the yield of doxorubicin was 21.5% greater than that of the parent strain. However, the higher fold enhancement reported in the literature may be due to the low yield of the original strain. Surprisingly, after deletion of the branch genes *dnrH* and *dnrX*, the yield of doxorubicin decreased instead, which is inconsistent with the findings of previous reports. The biomass of the engineered strain △H4 and △X1 decreased compared to that of the parental strain SIPI-7-14, from 15 to 11% and 12%, respectively. We speculated that the toxicity of doxorubicin is higher than the glycosylated product baumycin, which has a great effect on the growth of *S. peucetius*. The baumycin is sensitive to acid and can be easily converted into doxorubicin by adjusting the pH to 1.5–1.8, so it will not affect the doxorubicin yield. These results indicate that there is a significant difference in the results of genetic engineering modification between high-yielding strain and wild-type strain.

As the genetic background of strains changed after genetic engineering, it is necessary to optimize the medium or culture conditions to explore the production potential of engineered strains. This phenomenon has also been found in other strains(Li et al. 2022; Wan et al. 2023; Zhang et al. 2023b). RSM was widely used to design experiments and determine the optimal medium with fewer experimental trials and obtain more responses at the same time. The medium obtained through RSM method was better than previously reported single-factor optimization. The production of doxorubicin reached 1406 mg/L in the shake flask on the 7th day, which was 24.6% higher than before.

In conclusion, the yield of doxorubicin can be effectively improved by combination of resistance screening, genetic engineering and optimization of culture media, a 150.6% increase compared with the parent strain SIPI-14, which was the highest reported so far (Table 2). This will make it possible to produce doxorubicin through one-step fermentation instead of existing chemical semi-synthetic production method. Meanwhile, some other products derived from actinomyces fermentation can also use this strategy to improve the fermentation yield.

**Table 2:** Comparison of strategies and yields of doxorubicin-producing strains

Strain	Strategies	Reported production(mg/L)	References
*S. peucetius*	Doxorubicin-resistance selection, deletion gene *dnrU*,	1406 in shake flask	This study
△U1/*drrC*	overexpression resistance gene *drrC* and medium optimization	1461 in 10 L fermenter	
SIPI-11	Classical strain mutation and medium optimization	570 in shake flask, 1100 in 5 L fermenter	(Wang et al. 2018)
ATCC27952	Overexpression of *coaA* and *coaE* genes	3 in shake flask	(Lee et al. 2014)
ATCC27952	Overexpression of the global regulatory gene *afsR*	80 in shake flask	(Parajuli et al. 2005)
ATCC 29,050	Inactivation doxorubicin-modifying enzymes dnrX	41.3 in shake flask	(Lomovskaya et al. 1998)

### Electronic supplementary material

Below is the link to the electronic supplementary material.

Additional file: Table S1 Primers used in this study. Figure S1. Pathway for biosynthesis of doxorubicin. Figure S2 The vector maps of (a) pSET-△dnrU, (b) pSET-△dnrH, and (c) pSET-△dnrX used for deletion and (d) pSET152-*drrC*, (e) pSET152-*drrAB*, (f) pSET152-*drrD*, and (g) pSET152-*drrABC* expression of genes in *Streptomyces peucetius*. Table S2 Three-factor and three-level experimental design. Table S3 BBD design of process variables for experiment and values of experimental data for doxorubicin. Table S4 ANOVA for the response surface quadratic polynomial model

## Data Availability

All data generated or analyzed during this study are included in this article and its supplementary information file.
