# Correction to: Enhancement of doxorubicin production in *Streptomyces peucetius* by genetic engineering and process optimization

**DOI:** 10.1186/s13568-024-01724-1

**Published:** 2024-06-24

**Authors:** Songbai Yang, Jiali Gui, Zhengyu Zhang, Jiawei Tang, Shaoxin Chen

**Affiliations:** 1 National Key Laboratory of Lead Druggability Research, Shanghai Institute of Pharmaceutical Industry, China State Institute of Pharmaceutical Industry, 285 Gebaini Road, Pudong, Shanghai, 201203 P. R. China; 2 Department of Biological Medicines & Shanghai Engineering Research Center of Immunotherapeutics, Fudan University School of Pharmacy, 826 Zhangheng Road, Pudong, Shanghai, 201203 P. R. China

**Correction to: AMB Express (2024) 14:41** 10.1186/s13568-024-01699-z

Following publication of the original article (Yang et al. 2024), the authors regret for the affiliation errors.

The affiliation details for “Songbai Yang, Jiali Gui, Jiawei Tang and Shaoxin Chen” were incorrectly given as “National Key Laboratory of Lead Druggability Research, Shanghai Institute of Pharmaceutical Industry, State Institute of Pharmaceutical Industry, 285 Gebaini Road, Pudong, Shanghai 201203, P. R. China” but should have been “National Key Laboratory of Lead Druggability Research, Shanghai Institute of Pharmaceutical Industry, China State Institute of Pharmaceutical Industry, 285 Gebaini Road, Pudong, Shanghai 201203, P. R. China”.

The authors would like to apologise for any inconvenience caused.
