# Supplementary material for: Enhancement of doxorubicin production in Streptomyces peucetius by genetic engineering and process optimization
Source: AMB Express. 2024 Apr 24;14:41. doi: 10.1186/s13568-024-01699-z (PMC11043234; doi:10.1186/s13568-024-01699-z)
Supplement: Supplementary file 1 — Additional file: Table S1 Primers used in this study. Figure S1. Pathway for biosynthesis of doxorubicin. Figure S2 The vector maps of (a) pSET-△dnrU, (b) pSET-△dnrH, and (c) pSET-△dnrX used for deletion and (d) pSET152-drrC, (e) pSET152-drrAB, (f) pSET152-drrD, and (g) pSET152-drrABC expression of genes in Streptomyces peucetius. Table S2 Three-factor and three-level experimental design. Table S3 BBD design of process variables for experiment and values of experimental data for doxorubicin. Table S4 ANOVA for the response surface quadratic polynomial model [file 13568_2024_1699_MOESM1_ESM.docx]

Journal name: AMB Express

Manuscript Title: Enhancement of doxorubicin production in *Streptomyces peucetius* by genetic engineering and process optimization

Songbai Yang^1^, Jiali Gui^1^, Zhengyu Zhang^2^, Jiawei Tang ^1^, Shaoxin Chen ^1, *^

1: National Key Laboratory of Lead Druggability Research, Shanghai Institute of Pharmaceutical Industry, China State Institute of Pharmaceutical Industry, 285 Gebaini Road, Pudong, Shanghai 201203, P. R. China.

2: Department of Biological Medicines & Shanghai Engineering Research Center of Immunotherapeutics, Fudan University School of Pharmacy, 826 Zhangheng Road, Pudong, Shanghai 201203, P. R. China.

**Corresponding Author**

*** Shaoxin Chen:** National Key Laboratory of Lead Druggability Research, Shanghai Institute of Pharmaceutical Industry, China State Institute of Pharmaceutical Industry.

Address: 285 Gebaini Road, Pudong, Shanghai 201203, P. R. China.

E-mail: [sxzlb@263.net](mailto:sxzlb@263.net)

E-mails of Coauthors:

Songbai Yang：yangsongbai5588@163.com

Jiali Gui: 1035054767@qq.com

Zhengyu Zhang: zyzhang824@163.com

Jiawei Tang: 1257748979@qq.com

**Table S1** Primers used in this study

| **Primers** | **Sequence (5’-3’)** |
| --- | --- |
| **Primers for the construction of pSET-△d*nrU*, pSET-△*dnrH*, and pSET-△*dnrX*** | |
| *dnrU*-UP-F | GTCGACCTGCAGGGGGGGAAGCTTTTGCGCTGCATGGTCATC |
| *dnrU*-UP-R | TTGACCACGGGCGGCTACT |
| *dnrU*-DOWN-F | GAAGCCTGCGAGGTGATTG |
| *dnrU*-DOWN-R | CGCGCGGCCGCGGATCCTCTAGAGTACCACGCCCAGAAACGG |
| *dnrH*-UP-F | GTCGACCTGCAGGGGGGGAAGCTTCTCGGCGCAGGTCGTGCACCGC |
| *dnrH*-UP-R | GGGTGACCTGGGCGTAGACATGG |
| *dnrH*-DOWN-F | TGGACGGCCTGGACGTGGAGGTC |
| *dnrH*-DOWN-R | CGCGCGGCCGCGGATCCTCTAGAGTGCGGGGGCGAGTGAGACGGACG |
| *dnrX*-UP-F | GTCGACCTGCAGGGGGGGAAGCTTATCCCCGTTACCCCGATCTG |
| *dnrX*-UP-R | ATCAGCTGATCCCGGTGGCGTC |
| *dnrX*-down-F | GATCCCTCTCGATGCTGAGTGGC |
| *dnrX*-down-R | CGCGCGGCCGCGGATCCTCTAGACGTCTTGCGGCCGTTCAGGCGGT |
| **Primers for examining the genes *dnrU*, *dnrH* and *dnrX* deletion** | |
| △*dnrU* F | GCCGTCTCGACCCGCTTGGTCAGTGCGTCG |
| △*dnrU* R | TGTACCTGGAGGTAGATGCCGGGGTCCGTAC |
| △*dnrH* F | CTGGCTGACGCTCCACCAAC |
| △*dnrH* R | GCCGGTCACGATCACGGATC |
| △*dnrX* F | GTGGCTCGCCGCGACACTCGTCCC |
| △*dnrX* R | CCGACTTCACCGGTCTGGACGGCCC |
| **Primers for the construction of pSET-*drrC*, pSET-*drrAB*, pSET-*drrD* and pSET-drr*ABC*** | |
| *drrC* F | CCACAGGAGGACCCATATGATGCAGCAGCAGGCTATTCAGATCATCGG |
| *drrC* R | ACTAGTGGCGCGCCAAAGTTTTGTCTCAGCGCCGGGCCCGGTGGT |
| *drrAB* F | CCACAGGAGGACCCATATGGTGAACACGCAGCCGACACG |
| *drrAB* R | GGCGCGCCAAAGTTTTGTCTTCAGTGGGCGTTCTTGTTGC |
| *drrD* F | CCACAGGAGGACCCATATGGTGGCCCGGTACCCACAGGC |
| *drrD* R | GGCGCGCCAAAGTTTTGTCTCAGCTGATCCCGGTGGCGT |
| *drrAB* F (TY) | ACCACCGGGCCCGGCGCTGAGTGAACACGCAGCCGACAC |
| *drrAB* R(TY) | GGCGCGCCAAAGTTTTGTCTCAGTGGGCGTTCTTGTTGCG |
| **Primers for qRT-PCR** | |
| Q*drrC* F | GGATCATCCCGATCAAGGAGCGATT |
| Q*drrC* R | GTGTAGATGATGCCGTGTCCCTGGC |
| 16s *RNA* F | CCTTCGGGTTGTAAACCTCTTTCAGCA |
| 16s *RNA* R | CAACACCTAGTTCCCAACGTTTACGGC |

**Figure S1** Pathway for biosynthesis of doxorubicin([Niraula et al. 2010](#_ENREF_1))

**Figure S2** The vector maps of (a) pSET-△*dnrU*, (b) pSET-△*dnrH*, and (c) pSET-△*dnrX* used for deletion of genes and (d) pSET152-*drrC*, (e) pSET152-*drrAB*, (f) pSET152-*drrD*, and (g) pSET152-*drrABC* expression of genes in *Streptomyces peucetius*


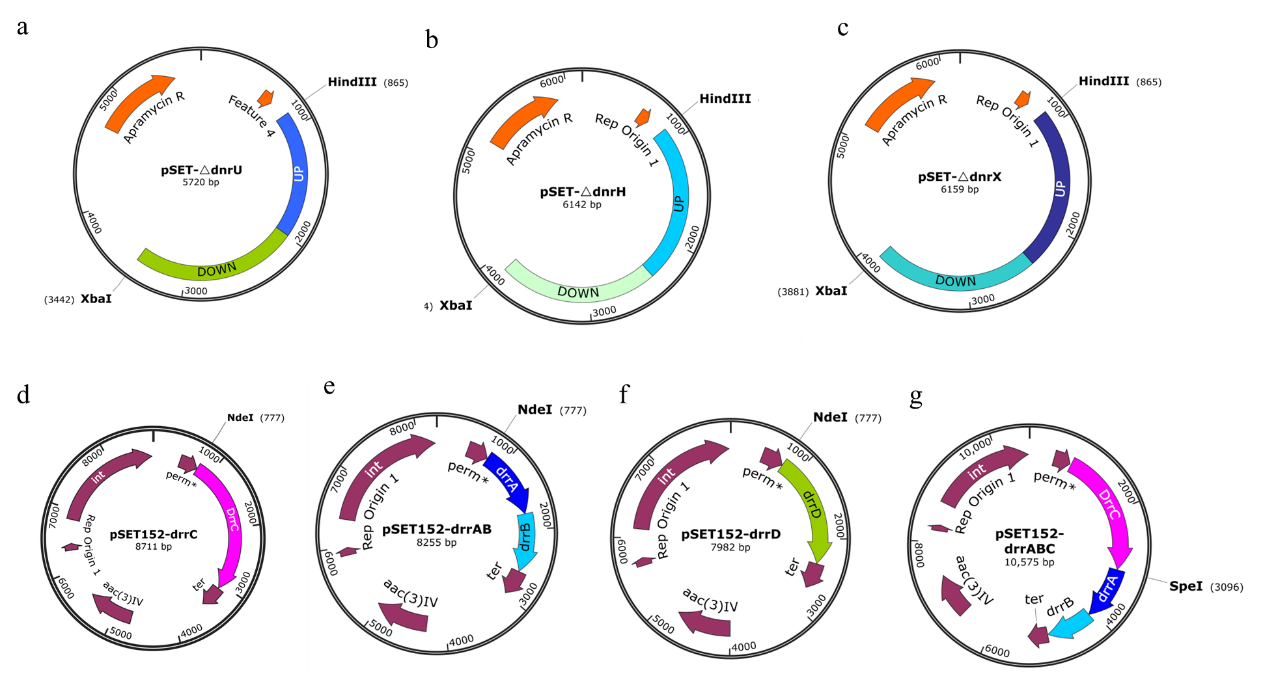


**Table S2** Three-factor and three-level experimental design

| **Factors（g/L） Codes level**  -1 0 +1 | | | | |
| --- | --- | --- | --- | --- |
| Maltodextrin | A | 110.0 | 120.0 | 130.0 |
| Dry yeast powder | B | 20.0 | 30.0 | 40.0 |
| Calcium chloride | C | 4.0 | 5.0 | 6.0 |

**Table S3** BBD design of process variables for experiment and values of experimental data for doxorubicin

| **Run** | **A** | **B** | **C** | **Doxorubicin（mg/L）** |
| --- | --- | --- | --- | --- |
| 1 | -1 | 0 | -1 | 921 |
| 2 | 0 | -1 | -1 | 1134 |
| 3 | 0 | 1 | 1 | 854 |
| 4 | 0 | 0 | 0 | 1165 |
| 5 | 1 | -1 | 0 | 903 |
| 6 | 1 | 1 | 0 | 1116 |
| 7 | 1 | 0 | 1 | 1333 |
| 8 | -1 | -1 | 0 | 1185 |
| 9 | 0 | -1 | 1 | 1148 |
| 10 | 0 | 0 | 0 | 1253 |
| 11 | 0 | 1 | -1 | 1171 |
| 12 | 0 | 0 | 0 | 1241 |
| 13 | 1 | 0 | -1 | 1190 |
| 14 | 0 | 0 | 0 | 1148 |
| 15 | -1 | 1 | 0 | 894 |
| 16 | -1 | 0 | 1 | 1182 |
| 17 | 0 | 0 | 0 | 1225 |

**Table S4** ANOVA for the response surface quadratic polynomial model

| Source | Sum of square | Degrees  of freedom | Mean square | F-value | P‑value Prob > F | Significant |
| --- | --- | --- | --- | --- | --- | --- |
| Model | 3.023E+005 | 9 | 33585.83 | 16.42 | 0.0006 | *** |
| A | 11026.12 | 1 | 11026.12 | 6.30 | 0.0533 |  |
| B | 2.384E+005 | 1 | 2.354E+005 | 116.55 | <0.0001 | *** |
| C | 40.50 | 1 | 40.50 | 0.020 | 0.8921 |  |
| AB | 1764.00 | 1 | 1764.00 | 0.86 | 0.3840 |  |
| AC | 210.25 | 1 | 210.25 | 0.10 | 0.7579 |  |
| BC | 1482.25 | 1 | 1482.25 | 0.72 | 0.4228 |  |
| A^2^ | 2047.17 | 1 | 2047.17 | 1.00 | 0.3504 |  |
| B^2^ | 48161.27 | 1 | 48161.27 | 23.55 | 0.0019 | ** |
| C^2^ | 290.06 | 1 | 290.06 | 0.14 | 0.7176 |  |
| Residual | 14317.45 | 7 | 2045.35 |  |  |  |
| Lack of Fit | 4890.25 | 3 | 1630.08 | 0.69 | 0.6032 | Not significant |
| Pure Error | 9427.20 | 4 | 2356.80 |  |  |  |
| Cor Total | 3.166E+005 | 16 |  |  |  |  |

*P < 0.05; **P < 0.01; ***P < 0.001; C.V.% =4.03 ;R^2^=0.9548 ; Adj R^2^ =0.8966 ; Pred R^2^ = 0.7063
